# Comparative Analyses of the Self-Sealing Mechanisms in Leaves of *Delosperma cooperi* and *Delosperma ecklonis* (Aizoaceae)

**DOI:** 10.3390/ijms21165768

**Published:** 2020-08-11

**Authors:** Linnea Hesse, Tim Kampowski, Jochen Leupold, Sandra Caliaro, Thomas Speck, Olga Speck

**Affiliations:** 1 Plant Biomechanics Group, Botanical Garden, Faculty of Biology, University of Freiburg, 79104 Freiburg, Germany; tim.kampowski@biologie.uni-freiburg.de (T.K.); sandra.caliaro@biologie.uni-freiburg.de (S.C.); thomas.speck@biologie.uni-freiburg.de (T.S.); olga.speck@biologie.uni-freiburg.de (O.S.); 2 Freiburg Materials Research Center (FMF), University of Freiburg, 79104 Freiburg, Germany; 3 Department of Radiology, Medical Physics, Medical Center, Faculty of Medicine, University of Freiburg, 79106 Freiburg, Germany; jochen.leupold@uniklinik-freiburg.de; 4 Cluster of Excellence *liv*MatS @ FIT Freiburg Center for Interactive Materials and Bioinspired Technologies, University of Freiburg, 79110 Freiburg, Germany

**Keywords:** ecological niche, damage management, leaf kinematics, morphology, biomechanics, self-repair, succulence, trichomes

## Abstract

Within the Aizoaceae, the genus *Delosperma* exhibits a vast diversification colonizing various ecological niches in South-Africa and showing evolutionary adaptations to dry habitats that might include rapid self-sealing. Leaves of *Delosperma* react to external damage by the bending or contraction of the entire leaf until wound edges are brought into contact. A study of leaf morphology and anatomy, biomechanics of entire leaves and individual tissues and self-sealing kinematics after a ring incision under low and high relative humidity (RH) was carried out comparing the closely related species *Delosperma cooperi* and *Delosperma ecklonis*, which are indigenous to semi-arid highlands and regions with an oceanic climate, respectively. For both species, the absolute contractions of the examined leaf segments (“apex”, “incision”, “base”) were more pronounced at low RH levels. Independent of the given RH level, the absolute contractions within the incision region of *D. cooperi* were significantly higher than in all other segments of this species and of *D. ecklonis*. The more pronounced contraction of *D. cooperi* leaves was linked mainly to the elastic properties of the central vascular strand, which is approximately twice as flexible as that of *D. ecklonis* leaves.

## 1. Introduction

The fast and efficient repair of damage, such as fissures, cuts and cracks, is a fundamental element of life. During a life-span, multiple internal and external factors can cause potentially critical damage or abrasions requiring maintenance and repair. As a result, an ability to self-repair, i.e., the autonomously sealing and healing of various forms of internal and external injuries, has evolved in all groups of living organisms ranging from single-celled bacteria to multicellular, hierarchically organized animals and plants [1,2,3,4,5,6,7,8]. In this context, the species-rich genus *Delosperma* (N.E.Br.) has proven to be a particularly suitable research object for comparative studies, not at least because of its high diversification. The hyperdiversification of Southern African ice plants belonging to the 1.13– to 6.49-million-years-old Ruschioideae lineage is associated with the preceding establishment of (semi-) arid environments within the region [9,10]. Valente et al. [10] have calculated a diversification rate of 4.4 species per million years, one of the fastest among the angiosperms, and have shown correlations between the aridity and radiation of the Aizoaceae. In addition, they have found a significant relationship between the richness of the core ruschioid genus and precipitation, temperature and increasing topographical complexity, leading to the hypothesis of “ecological opportunities” as the driving force of the described diversification. Another hypothesis postulates that climatic or ecological factors alone cannot explain the remarkable speciation burst and, thus, Klak et al. [9] propose that morphological and anatomical “key innovations” such as the wide-band tracheids, the cylindrical or trigonous leaf shape and hygrochastic capsules have facilitated the major radiation of the core Ruschioideae. In addition to preventing cavitation, Mauseth [11] and Arruda and Melo-de-Pinna [12] have interpreted the wide-band tracheid as a case of paedomorphosis.

As *Delosperma* species are mostly endemic to hot semi-arid to arid climates, their leaves reveal several morphological and anatomical adaptations to dry habitats, such as the development of succulent water-storing tissues. The stored water can be utilized for photosynthesis or growth during drought periods [13,14]. Since injuries to the leaves can cause severe drought stresses, a fast and reliable self-repair mechanism is a major selection advantage. In the leaves of *Delosperma cooperi* (Hook.f.), a rapid self-sealing phase initiates self-repair and restores the functional integrity of the leaf preventing any further loss of stored water from the system. Subsequently, a comparatively slow self-healing phase restores structural integrity and, hence, in part the mechanical properties of the leaf [8,15,16]. The quick initial self-sealing phase is of particular interest, as it involves a deformation of the entire leaf rapidly bringing the wound edges into contact and preventing further water loss. This distinguishes *D. cooperi* from other plants investigated to date in which self-sealing is accomplished via for example (1) the deformation of dermal tissues, (2) turgescent parenchyma cells expanding into (micro-)fissures, (3) the leakage of plant sap such as mucilage or latex into the incision gap or (4) in combination with self-healing, the formation of callus tissues or the coagulation of lattices in latex-bearing plants [5,7,17,18,19].

The deformation of *D. cooperi* leaves during self-sealing differs dependent on the type of wound applied to the leaf. If a longitudinal or transversal cut is applied unilaterally, i.e., only on one side of the leaf, the leaf will bend towards the side of the incision leading to the sealing of the wound [15,16]. If an injury is applied around the circumference of the leaf, the entire leaf will perform a contraction along the leaf axis [15,16]. The findings of Dumais and Forterre [20] (also see Skotheim and Mahadevan [21]) suggest two mechanisms acting as possible driving forces behind the self-sealing kinematics: hydraulic movements or mechanical instabilities. Analytical [4] and numerical [15] models have revealed that both mechanical instabilities and hydraulic movements cause the self-sealing deformation of *D. cooperi* leaves. The mechanical instabilities within the leaf result from growth processes and increased water storage during leaf ontogeny, alternatingly pre-stressing some of the leaf tissues in tension (including the central vascular strand, the net of peripheral vascular bundles with wide-band tracheids and the epidermis) and others in compression (chlorenchyma and hydrenchyma). This pre-stressed state of equilibrium is disturbed upon wounding and the stored energy is released, initially opening the incision during the first three to five minutes. In addition, the wound edges show a rolling-in within a few minutes after the damage. Subsequently, water evaporates from the incision region, reducing the compressive stresses of the parenchyma tissues within the entire leaf as global water flow compensates for the loss of water from leaf tissues in close proximity to the incision. This hydraulic process is predominantly governed by the permeability of tissues and the reflection coefficient (a measure for the selectivity of a membrane towards a given solute) [15]. During this self-sealing phase of about 60 min, the wound edges are brought together because of relaxation of the compressive and tensile stresses in the individual tissues. This relaxation leads to a local (incision region) and a global (whole leaf) contraction of the leaf. The mechanical integrity of the leaf during the entire self-sealing process is ensured by a mechanical state of equilibrium in which compressive stress (parenchymal tissue) and tensile stress (vascular tissue and epidermis) are almost equal [15].

In general, the diversity of self-repair processes found in biological systems can be a source of inspiration for the development of novel concepts for damage management and self-repair in technological systems [5,6,8,22,23,24,25,26,27]. In particular, the self-repairing mechanisms of the succulent leaves of the ice plant *D. cooperi* [4,5,8,15,16] have served as a biological role model for a novel bio-inspired self-healing polymer with shape memory effect [28].

Although some investigations and modeling have previously been carried out on *D. cooperi*, various scientific questions remain that have been answered in the framework of the present study comparing two closely related *Delosperma* species both native to South-Africa. *D. cooperi* grows in mountain regions with mean altitudes of 1419 m and diurnal and seasonal fluctuations of drought stress. *Delosperma ecklonis* (Schwantes) is native to ocean regions with mean altitudes of 283 m and experiences evenly distributed yearly rainfall and consistently higher relative humidity (RH) than does *D. cooperi*, which has seasonally variable rainfall. The central objective of this study has been to compare the two *Delosperma* species against the background of adaptations to their different habitats with respect to (1) leaf morphology and anatomy, (2) the biomechanical properties of entire leaves and individual tissues, and (3) self-sealing kinematics after a ring incision at low and high RH.

## 2. Results

### 2.1. Habitus and Leaf Morphology

**Figure 1:**
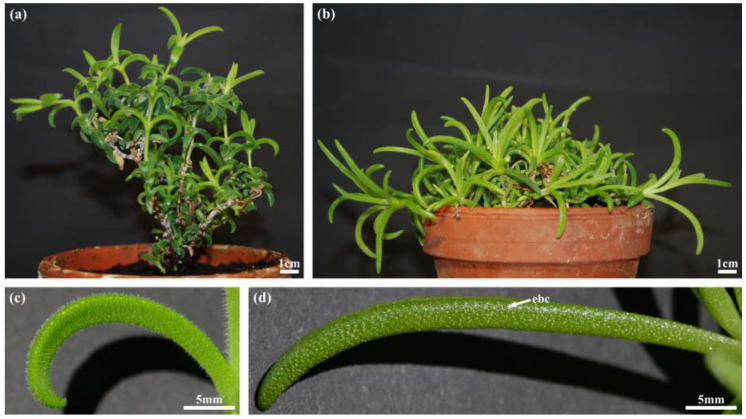
Comparison of the habitus (**a**,**b**) and leaf morphology (**c**,**d**) of *D. ecklonis* (**a**,**c**) and *D. cooperi* (**b**,**d**). (**a**) The habitus of *D. ecklonis* is characterized by erect branches. (**b**) Branches of *D. cooperi* are decumbent. (**c**) Leaves of *D. ecklonis* are triquetrous, pubescent and recurved. (**d**) Semi-terete to terete glabrous leaves with rounded papillary epidermal bladder cells (ebc; arrow) are characteristic for *D. cooperi*.

*D. ecklonis* and *D. cooperi* are richly branched shrubs with succulent leaves (Figure 1). Younger branches of *D. ecklonis* are mainly erect, hairy (velvety) and whitish whereas older branches are brownish scaly (Figure 1a). In comparison, young branches of *D. cooperi* are erect and green and become smooth, ochre and decumbent when older (Figure 1b). Individual plants of *D. ecklonis* are tall (height: 129.6 ± 27.2 mm) when compared with *D. cooperi* (height: 68.0 ± 13.7 mm). The corresponding diameter of the plants, however, is comparable (149.5 ± 57.0 mm for *D. ecklonis* and 150.0 ± 44.0 mm for *D. cooperi*).

**Figure 2:**
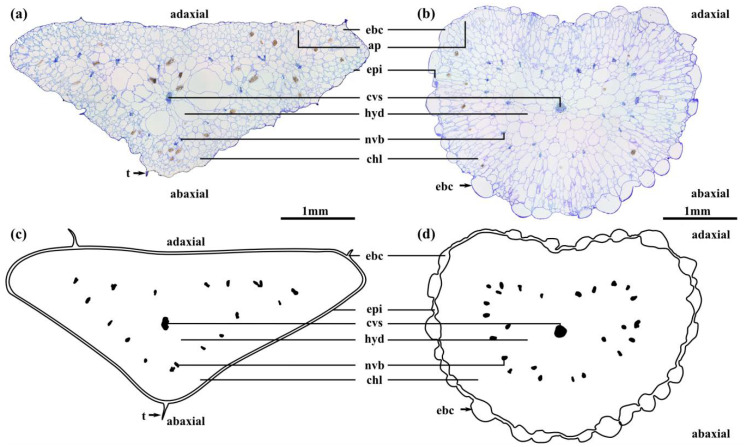
Comparison of transverse sections of the leaves of *D. ecklonis* (**a**) and *D. cooperi* (**b**). (**a**) Light microscopic image of the transverse section of a triquetrous *D. ecklonis* leaf. Large hydrenchyma cells (hyd) surround the central vascular strand (cvs) and can be clearly distinguished from the smaller chloroplast-rich chlorenchyma tissue (chl). Trichomes (t) show bladder-like enlargements at the leaf surface. (**b**) Light microscopic image of a transverse section of a terete *D. cooperi* leaf with slightly canaliculated adaxial and convex abaxial sides. Schematic drawings of a transverse section of leaves of *D. ecklonis* (**c**) and *D. cooperi* (**d**) highlighting the various tissue layers. ap: air pockets; chl: chlorenchyma; cvs: central vascular strand; ebc: epidermal bladder cell; epi: epidermis; hyd: hydrenchyma; nvb: peripheral net of vascular bundles; t: trichome.

**Figure 3:**
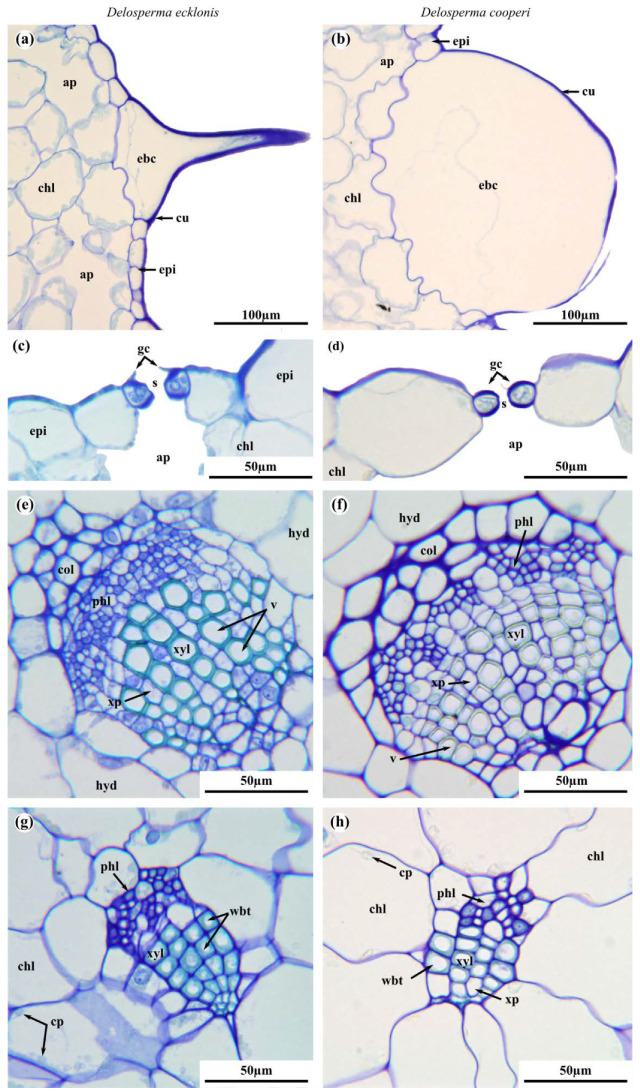
Comparison of epidermal idioblasts and the vasculature of the leaves of *D. ecklonis* (**a**,**c**,**e**,**g**) and *D. cooperi* (**b**,**d**,**f**,**h**). (**a**) Basally thickened and distally elongated (trichome-like) epidermal bladder cell (ebc) of *D. ecklonis*. (**b**) Epidermal bladder cells (ebc) of *D. cooperi* are large and rounded. Longitudinal section of a stoma (s) of *D. ecklonis* (**c**) and *D. cooperi* (**d**) with guard cells (gc) and air pockets (ap). Central vascular strand of *D. ecklonis* (**e**) and *D. cooperi* (**f**) with angular collenchyma (col) cap on the phloem (phl) side of the bundle. (**g**) Peripheral vascular bundles of *D. ecklonis* are located in close proximity to the chlorenchyma (chl) and are characterized by wide-band tracheids (wbt). (**h**) Wide-band tracheids are also found in peripheral vascular bundles of *D. cooperi*. ap: air pocket; chl: chlorenchyma; col: collenchyma; cp: chloroplast; cu: cuticula; ebc: epidermal bladder cell; epi: epidermis; gc: guard cell; hyd: hydrenchyma; phl: phloem; s: stoma; t: trichome; v: vessel element; wbt: wide-band tracheids; xp: xylem parenchyma; xyl: xylem.

**Figure 4:**
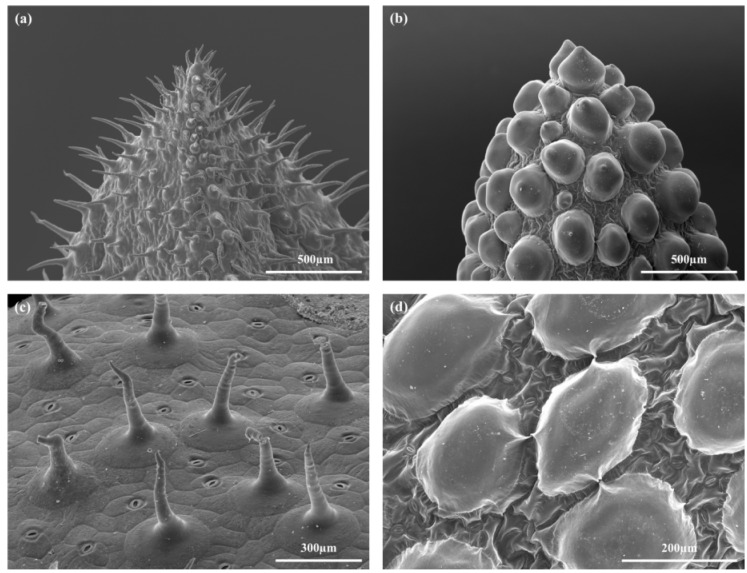
Scanning electron microscope images of leaf tips (**a**,**b**) and epidermal bladder cells (**c**,**d**) of *D. ecklonis* (**a**,**c)** and *D. cooperi* (**b**,**d**).

The morphology of the leaves of the two species differs greatly. *D. ecklonis* leaves are triquetrous, pubescent and recurved with a slightly concave upper surface and a convex abaxial side (Figure 1c and Figure 2a,c). Having a length of 27.4 ± 26.5 mm, they are much shorter than the leaves of *D. cooperi* (length = 45.5 ± 1.9 mm; [15]). Leaves of *D. cooperi* are glabrous, semi-terete to terete with a slightly canaliculated upper surface and a convex abaxial side (Figure 1d and Figure 2b,d). The trichomes of *D. cooperi* are developed to rounded papillary epidermal bladder cells (ebc), which are macroscopically visible on the leaf surface (Figure 1d, Figure 2b, Figure 3b and Figure 4d). Leaves of both species were tapered on the tip (Figure 1c,d).

### 2.2. Leaf Anatomy

**Figure 5:**
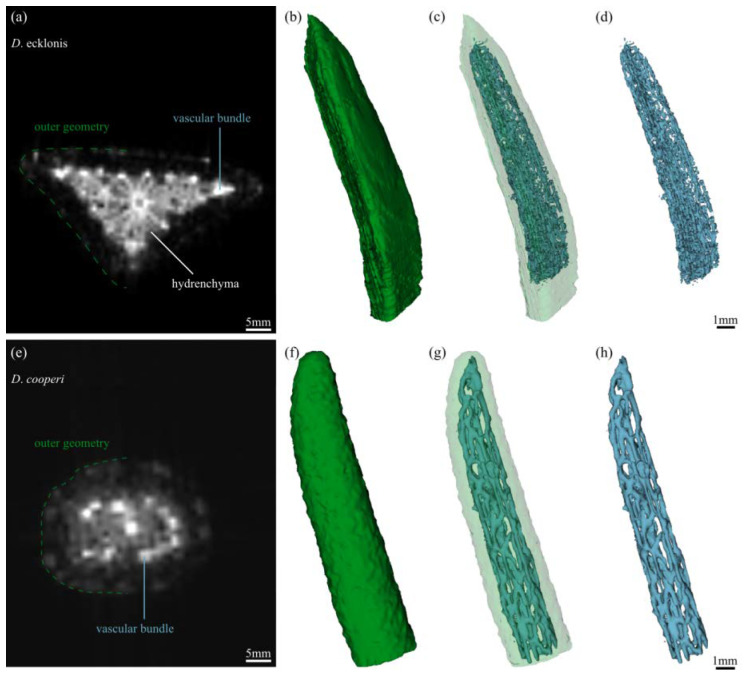
Magnetic resonance imaging of the leaf tips of *D. ecklonis* (**a**–**d**) and *D. cooperi* (**e**–**h**). (**a**) 2D tomogram (cross-section) of a *D. ecklonis* leaf. The contrasting agent (MnCl) does not provide sufficient contrast between large-celled hydrenchyma tissue and vascular bundles. (**b**–**d**) Quasi 3D data representation of the outer leaf geometry (**b**,**c**) and inner 3D vascular system (**d**) of the leaf of *D. ecklonis*. The low tissue contrast between the hydrenchyma and vascular bundles results in the reduced quality of the segmentation of the complex vascular network of the leaf in comparison with the results for *D. cooperi*. (**e**) 2D tomogram (cross-section) of a *D. cooperi* leaf. Bright appearance of vascular system by MnCl contrasting. (**f**–**h**) Quasi 3D data representation of the outer leaf geometry (**f**,**g**) and inner 3D vascular system (**h**) of the leaf of *D. cooperi*. The dotted line highlighting the outer geometry in (**a**,**e**) does not fully outline the entire leaf to allow better visualization of the image quality. Vascular anastomosis is frequent along the entire axis of the leaf within peripheral vascular bundles and between peripheral bundles and the central vascular strand.

The leaves of *D. ecklonis* and *D. cooperi* were comprised of five consecutive tissue layers from the outside inwards: the epidermis, the chlorenchyma, the peripheral net of vascular bundles, the hydrenchyma and the central vascular strand (Figure 2). Two epidermis idioblast types (epi in Figure 2) can be detected: the guard cells of the stomata (gc in Figure 3c,d) and the epidermal bladder cells (ebc in Figure 2 and Figure 3a,b). The epidermal bladder cells of *D. ecklonis* are thickened basally but grow out to form slim trichomes distally (Figure 2a, Figure 3a and Figure 4a,c). This differs from the rounded and papillose, large and densely packed epidermal bladder cells of *D. cooperi* (Figure 2b, Figure 3b and Figure 4b,d). A thick and structurally inconspicuous cuticula covers the leaves. Chloroplasts allow the chlorenchyma to be distinguished from the inner chloroplast-free hydrenchyma tissue. A comparison of the aspect ratios (calculated from cross-sectional diameters) of chlorenchyma and hydrenchyma cells reveals significant differences for *D. cooperi* with median values of 1.54 for the chlorenchyma and 1.26 for the hydrenchyma (Wilcoxon Mann–Whitney signed rank test, *W* = 542, *p* < 0.05). In contrast, no significant differences have been found for *D. ecklonis* with median aspect ratios of 1.50 for chlorenchyma cells and 1.41 for hydrenchyma cells (Wilcoxon Mann–Whitney signed rank test, *W* = 108, *p* > 0.05) (see Supplementary Data; 10.5281/zenodo.3885176). Large air pockets (ap in Figure 2a,b) can be found within the chlorenchyma of both species and are closely associated with the stomata of the leaves. The hydrenchyma cells of *D. ecklonis* with cell diameters ranging from 22 to 412 µm are noticeably larger than those of *D. cooperi* with cell diameters between 20 and 228 µm (compare hyd in Figure 2a,b; see Supplementary Data; 10.5281/zenodo.3885176). The vascular system of *D. ecklonis* and *D. cooperi* was comprised of an axially continuous central vascular strand (cvs in Figure 2, Figure 3c,g and Figure 5a,e) and a peripheral net of vascular bundles (nvb in Figure 2 and Figure 5a,e) located at the border between the chlorenchyma and hydrenchyma, as common for Aizoaceae with hydrenchyma tissue (“storage-succulence”; [14,29,30]). The leaves of both species are endoscopic with xylem tissue directed towards the leaf center (also see [30]). Wide-band tracheids (wbt in Figure 3g,h) characterize peripheral vascular bundles, which are in close proximity to the chlorenchyma tissue. Vascular bundles located within the hydrenchyma lack wide-band tracheids (Figure 3), irrespective of whether it is the central vascular strand or a vascular anastomosis towards the peripheral vascular network. The diameter of the central vascular strand is in general larger than those of the individual peripheral vascular bundles (compare Figure 3e,f with Figure 3g,h, respectively). A cap of angular collenchyma is located at the phloem side and rarely at the xylem side of the central vascular strand but is missing in peripheral vascular bundles (compare Figure 3e,f with Figure 3g,h, respectively).

### 2.3. Leaf Morphometry

The leaves of *D. ecklonis* and *D. cooperi* significantly varied in length and diameter (Table 1). Moreover, the latter measurement could be divided into a minor and a major axis with significantly varied sizes in both species (Table 1). In general, the different geometries of the triquetrous *D. ecklonis* leaves and the semi-terete to terete *D. cooperi* leaves were well reflected by their internal morphology, which revealed that comparable tissues were almost always of significantly different thickness between the two species (Table 1). In particular, the thicknesses of both the chlorenchyma and the hydrenchyma were more pronounced in leaves of *D. ecklonis*, whereas the opposite was true for the thicknesses of the epidermis and the epidermal bladder cells, which were thicker in *D. cooperi* leaves. Only the thicknesses of the central vascular strand and the net of vascular bundles (measured along the major leaf diameter) were of comparable size between the two species (Table 1).

In *D. cooperi*, most of the tissues had a constant thickness throughout the cross-section (i.e., both along the minor and major diameters) of the leaf (Table 1). However, the thickness of its central vascular strand was axis-dependent (Table 1). The results for *D. cooperi* are taken from Klein et al. [15]. Contrastingly, the majority of tissue thicknesses measured for *D. ecklonis* exhibited an axis dependency (Table 1). Only the thicknesses of the epidermis and the epidermal bladder cells were axis-independent (Table 1).

### 2.4. Leaf Biomechanics

#### 2.4.1. Elastic Modulus and Tensile Strength

The mechanical properties of the entire leaf and of single tissue layers were measured in tensile tests that provided the basis for calculations of elastic modulus and tensile strength (Table 2). Measurements for the epidermis of *D. ecklonis* in transverse and longitudinal directions yielded no significant differences, either for the elastic modulus or for the tensile strength (Table 2). In *D. cooperi*, the elastic modulus of the epidermis was also not significantly different in transverse and longitudinal directions, whereas the tensile strength differed significantly (Table 2).

#### 2.4.2. Elastic Modulus of Parenchymatous Tissues

The elastic moduli of the hydrenchyma and chlorenchyma were calculated on the basis of an equation suggested by Nilsson et al. [31]. For *D. ecklonis*, median cell diameters (*d_c_*) of 84.73 µm and 73.72 µm and median cell wall thicknesses (*t_cw_*) of 0.37 µm and 0.35 µm were measured for the hydrenchymatic and chlorenchymatic cells, respectively. The parenchymatic cells of *D. cooperi* exhibited median diameters of 92.00 µm (hydrenchyma) and 62.50 µm (chlorenchyma) and had median equally thick cell walls (0.42 µm). By taking into account the measured turgor median values of *P* = 0.05 MPa for *D. ecklonis* and of *P* = 0.04 MPa for *D. cooperi* and the median Poisson’s ratios of *v* = 0.29 for *D. cooperi* and of *v* = 0.35 for *D. ecklonis* and by using an elastic modulus of cell walls of *E_cw_* = 5.00 MPa [32], elastic moduli could be calculated for both hydrenchyma and chlorenchyma in terms of both species (Table 2).

#### 2.4.3. Poisson’s Ratio and Turgor

Poisson’s ratios from entire leaves of *D. cooperi* and *D. ecklonis* were calculated from images taken during tensile tests. The values differ significantly between the two species (Table 2). In addition, no correlation between the turgor of single cells and their position in the leaf could be found in either species (Spearman’s rank-order correlation, Spearman’s *ρ* = −0.29). The turgor measured in the leaves of the two *Delosperma* species did not differ significantly (Table 2).

#### 2.4.4. Leaf Kinematics

**Figure 6:**
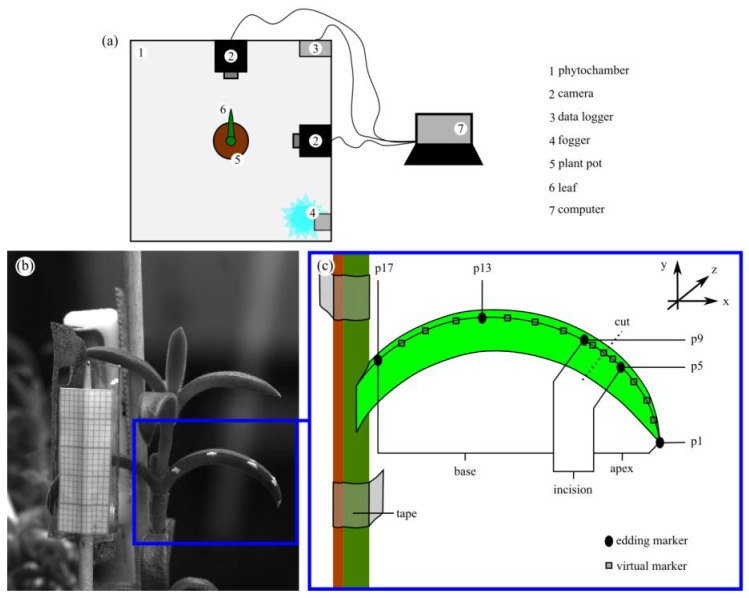
Video tracking of the self-sealing kinematics of *D. ecklonis* leaves. (**a**) Experimental setup within a temperature and relative humidity (RH) controlled custom-built phytochamber. (**b**) Image of a *Delosperma* leaf and sample mounting during an experiment. The stem is fixed to a bamboo stick by using tape. The white marker points indicate the apex (p1), the regions 2 mm above (p5) and below (p9) the incision (cut), the base (p17), and half the distance between points p9 and p17 (p13) of the leaf. (**c**) Schematic representation of the sample mounting and tracker placement along a leaf. The leaf segments “apex”, “incision” and “base” are indicated.

**Figure 7:**
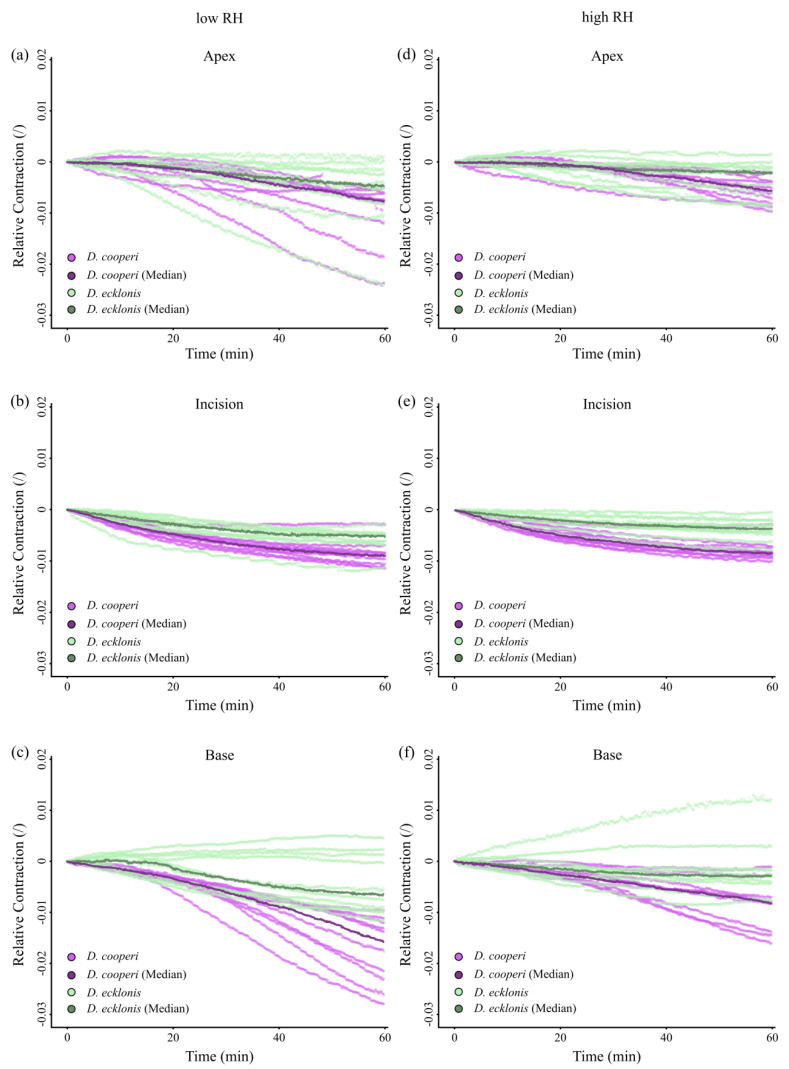
Relative values of self-sealing kinematics of *D. cooperi* and *D. ecklonis* at two different humidity levels (RH). (**a**–**c**) Relative contractions of leaf segments (apex, incision region and base) at low RH (*D. cooperi*: 44.0%; *D. ecklonis*: 43.6%) over time. (**d**–**f**) Relative contractions of leaf segments (apex, incision region and base) at high RH (*D. cooperi*: 59.6%; *D. ecklonis*: 57.3%) over time. Each graph depicts the ten individual measurements (light colors) and their corresponding median lines (dark colors) for both *D. cooperi* (purple curves) and *D. ecklonis* (green curves).

**Figure 8:**
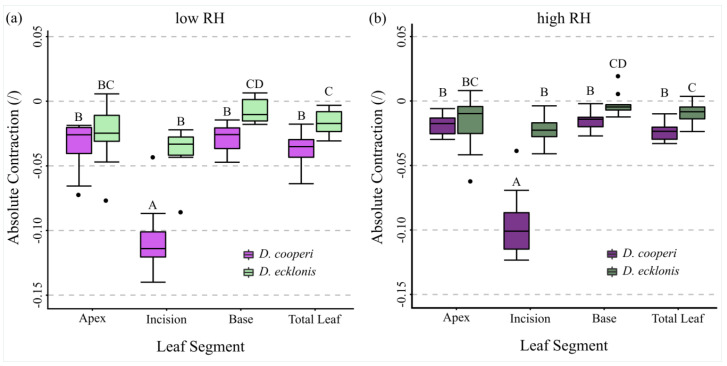
Statistical analyses of the self-sealing kinematics of *D. cooperi* and *D. ecklonis* at two different RH levels. Segment-specific comparison of the absolute contractions for both *D. cooperi* and *D. ecklonis* seen at (**a**) low RH and (**b**) high RH. Each boxplot resembles a sample size of *n* = 10. Statistical annotations are given above each boxplot. Light-colored and dark-colored boxplots represent data measured at low (*D. cooperi*: 44.0%; *D. ecklonis*: 43.6%) and high (*D. cooperi*: 59.6%; *D. ecklonis*: 57.3%) RH levels, respectively. Purple boxplots indicate the leaf kinematics of *D. cooperi*, whereas green boxplots highlight the results of *D. ecklonis*.

**Figure A1:**
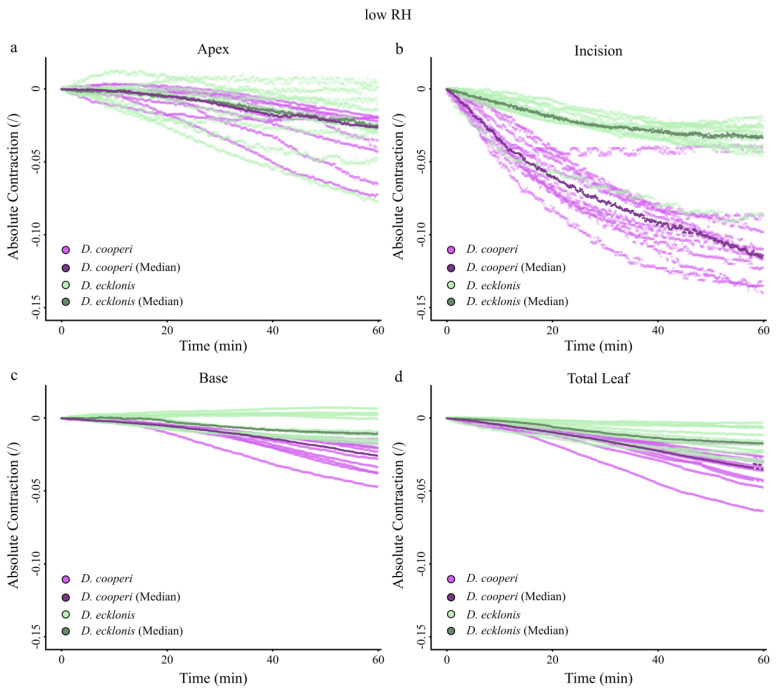
Absolute values of self-sealing kinematics of *D. cooperi* and *D. ecklonis* at low relative humidity (RH). Absolute contraction of (**a**) apical leaf segments, (**b**) leaf segments at the incision region, (**c**) basal leaf segments and (**d**) total leaves over time. On average, the relative humidities were 44.0% (IQR: 1.5%) and 43.6% (IQR: 0.9%) for *D. cooperi* and *D. ecklonis*, respectively. Each graph depicts the ten individual measurements (light colors) and their corresponding median lines (dark colors) for both *D. cooperi* (purple curves) and *D. ecklonis* (green curves).

**Figure A2:**
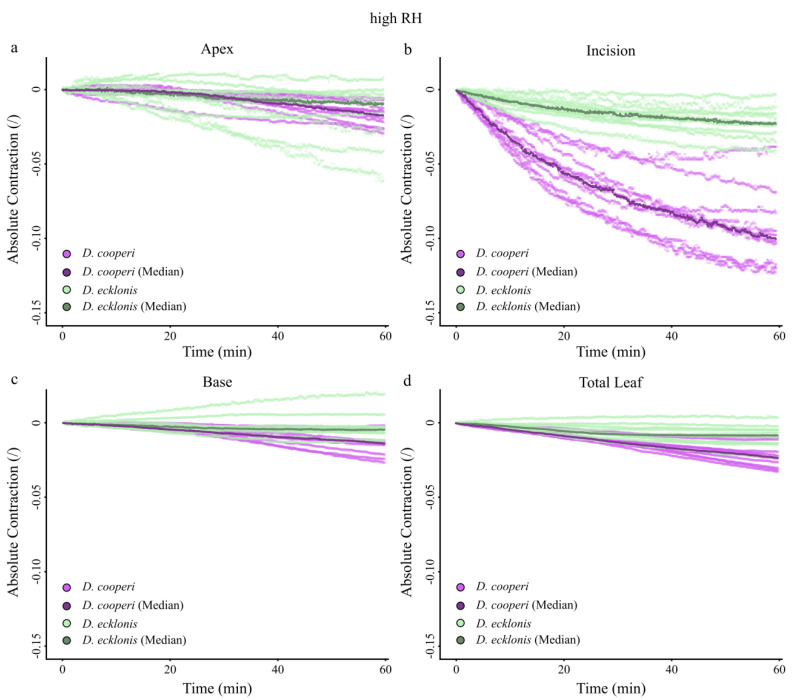
Absolute values of self-sealing kinematics of *D. cooperi* and *D. ecklonis* at high relative humidity (RH). Absolute contraction of (**a**) apical leaf segments, (**b**) leaf segments at the incision region, (**c**) basal leaf segments and (**d**) total leaves over time. On average, the relative humidities were 59.6% (IQR: 1.2%) and 57.3% (IQR: 1.0%) for *D. cooperi* and *D. ecklonis*, respectively. Each graph depicts the ten individual measurements (light colors) and their corresponding median lines (dark colors) for both *D. cooperi* (purple curves) and *D. ecklonis* (green curves).

With the exception of the apical leaf segments, the absolute and relative contractions of individual leaf segments (“apex”, “incision” and “base” in Figure 6c) were more pronounced in *D. cooperi* when compared with *D. ecklonis*, irrespective of whether the kinematic experiments were carried out at low or high RH. The highest species-specific kinematical differences occurred at the incision region (Figure 7 and Figure 8 and Figure A1 and Figure A2 in Appendix A).

For both *D. cooperi* and *D. ecklonis*, the absolute contractions of leaf segments were more prominent at low RH levels than at high RH levels (Figure 8 and Figure A1 and Figure A2 in Appendix A, Supplementary Video Svideo1: 10.5281/zenodo.3885166 and SVideo2: 10.5281/zenodo.3885174). However, independent of the given RH level, the absolute contractions within the incision regions of *D. cooperi* were significantly higher than those of all other segments (including those of *D. ecklonis*) (Scheirer-Ray-Hare Test, low RH level: *F*_3,72_ = 21.13, *p* < 0.001, high RH level: *F*_3,72_ = 17.41, *p* < 0.001, pairwise Wilcoxon post-hoc testing, Figure 8a,b). Compared with *D. cooperi* the segment-specific differences in leaf kinematics were less pronounced in *D. ecklonis* at both RH levels, although the absolute contraction of the incision region and the basal segment for a given level of RH was still highly significantly different in both species (Scheirer-Ray-Hare Test, *D. cooperi*: *F*_3,72_= 45.24, *p* < 0.001, *D. ecklonis*: *F*_3,72_ = 13.57, *p* < 0.001, pairwise Wilcoxon post-hoc testing, Figure 8a,b).

## 3. Discussion

According to Hartmann [35], the genus *Delosperma* is endemic to regions that are characterized by rainy summers and dry winters, mainly in South Africa, East Africa and the Arabian Peninsula, and colonizes various habitats within these regions. The climate data for *D. cooperi* match the general description for the genus, as it is exposed to drought stress induced by seasonal fluctuations of monthly precipitation, which is not the case for *D. ecklonis* (Figure 10). In addition, the annual RH is, in general, lower for *D. cooperi* (ca. 20%) (Figure 10) and data concerning the daily fluctuation of RH indicate additional drought stresses experienced by this species (daily fluctuations of ca. 40% RH) in comparison with *D. ecklonis* (daily fluctuations of ca. 20%) (Table A1; note the small sample number of *n* = 2). Hence, *D. cooperi* colonizes mountain regions with mean altitudes of 1419.2 m and daily and seasonal fluctuations of drought stress, whereas *D. ecklonis* is found in ocean-bordering regions (mean altitude of 283.1 m) with a comparably constant precipitation and RH throughout the year. These differences in habitat and climate of the two species are reflected in the different adaptations that they have developed during biological evolution of (1) the morphology, (2) the anatomy and (3) the wound reaction (self-sealing) of the leaves (see Table 3 for a summary).

Leaves of *Delosperma* are commonly fleshy and “soft to touch” [35], showing multiple morphological and anatomical traits linked to leaf succulence. Both *D. cooperi* and *D. ecklonis* have morphological-anatomical characteristics typical for “storage succulence” (also partial or storage-cell succulence), in which the task of assimilation and water storage are divided between an outer chlorenchyma layer and a central hydrenchyma tissue, respectively. In contrast, “all-cell succulence” describes the situation in which a single tissue performs both water-storage and photosynthesis, as found, for example, in *Crassula* species [30,36,37]. In general, the storage of water in succulent plants allows them to be temporarily independent from the external water supply during droughts and leads to an increase in organ volume. In *Delosperma*, the storage of water within the hydrenchyma tissue of the leaves increases leaf volume resulting in a thickened leaf morphology. According to Ogburn and Edwards [37], this results in a transport challenge within the leaves imposed by the mesomorphic leaf geometry. The enlarged leaf volume increases the hydraulic path-length between the vascular bundles and peripheral photosynthetic tissues. In unifacial leaves of many leaf-succulent plants this challenge is coped with by the development of 3D venation patterns [30,37,38]. Both *D. cooperi* and *D. ecklonis* have developed a 3D vascular system with a central vascular strand (cvb in Figure 2) and an endoscopic (phloem pointing towards the epidermis) peripheral network of vascular bundles. The majority of cells in the xylem of peripheral vascular bundles are wide-band tracheids, which are located at the boundary of the chlorenchyma and hydrenchyma (Figure 2, Figure 3 and Figure 5) [30,37,39]. These wide-band tracheids (wbt in Figure 3) are considered as to be specialized water transport cells representing an evolutionary adaptation to drought stress by preventing cell collapse during desiccation and enabling the reversible additional storage of water during water uptake. Although wide-band tracheids provide only limited resistance to tensile and compressive stress because of their annular secondary thickenings, Bobich and Nobel [40] hypothesize that these thickenings reduce shear between the cells, because the secondary thickenings of adjacent vascular tracheids usually alternate and create an interlocking of their lateral walls. Wide-band tracheids are only found in a few taxa, such as the Portulacaceae, Cactaceae and Aizoaceae, and in the latter within mainly the core Ruschioideae to which *Delosperma* belongs [9,14,30,37,39]. In particular, in the context of a rapid motion-based self-sealing of wounds in *Delosperma* leaves, the mechanical properties of wide-band tracheids play a decisive role [4]. Despite these similarities, the leaves of *D. ecklonis* and *D. cooperi* reveal several morphological, anatomical and biomechanical differences.

**Figure 9:**
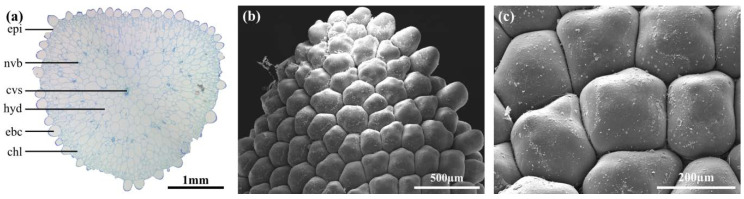
*Delosperma harazianum.* (**a**) Light microscopic image of a transverse section of an almost circular leaf stained with toluidine blue. (**b**,**c)** Scanning electron microscope images of a leaf tip (**b**) and epidermal bladder cells (**c**). chl: chlorenchyma; cvs: central vascular strand; ebc: epidermal bladder cell; epi: epidermis; hyd: hydrenchyma; nvb: peripheral net of vascular bundles.

Terete leaves with a circular or oval cross-section have, compared with leaves having a triangular cross-section, a minimized surface area (sa) to volume (vol) ratio (sa/vol) and can be considered as an advanced evolutionary adaptation to arid environments as they (1) maximize their water storage capacity and (2) reduce transpirational water loss by reducing their leaf surface area [9,30,37,38]. Furthermore, the leaves of *D. ecklonis* are pubescent, i.e., they have trichomes sitting at the tip of short papillae or epidermal bladder cells, whereas the glabrous leaves of *D. cooperi* are covered with rounded epidermal bladder cells (ebc in Figure 2 and Figure 3). Bladder cells are known to occur in various halophytes, such as the Chenopodioideae, Oxalidaceae and Mesembryanthemoideae, and contribute to succulence by serving as additional peripheral water storage or by contributing to salt tolerance [41,42]. Agarie et al. [41] have shown that wild-types *Mesembryanthemum crystallinum* (L.; Aizoaceae), which possess bladder cells, have increased leaf succulence, leaf water content and a 1.5-fold higher salt content compared with a mutant without bladder cells. Hence, the modification of trichomes to epidermal bladder cells can also be seen as an advanced evolutionary adaptation to drought stress in *D. cooperi* by increasing succulence and salt tolerance. The development of pronounced epidermal bladder cells is, in general, common for *Delosperma* species native to semi-arid to arid regions with seasonal fluctuations of drought stress, such as species from Gauteng (see Figures 19–21 in [43]). This also applies to *D. harazianum* (Deflers), which is endemic to mountain regions (altitudes of around 2500 m, unpublished data and [44]; Figure 9) of the Arabian Peninsula, Yemen [35], and which belongs to the Köppen-Geiger climate classification of BWh (“arid, desert, hot = average annual temperature above 18 °C“; [45,46]). Consideration of the species-specific climates, the density and shape of epidermal bladder cells and the geometry of the leaves reveals a transition: *D. ecklonis*, which grows in an ocean climate (Cfb), exhibits trichomes on leaves with a triangular cross-section (Figure 4a,c). *D. cooperi*, which is native to a dry-winter subtropical highland climate (Cwb), has leaves with an oval cross-section and individual, spaced epidermal bladder cells (Figure 4b,d). *D*. *harazianum*, which is native to a hot desert climate (Bwh) reveals an almost circular cross-section of leaves and epidermal bladder cells are densely packed and round (Figure 9). The adaptation to increasingly dry climatic conditions is also mirrored by the shape of the leaves. The leaves of *D. ecklonis* with their irregular tetrahedral shape have an sa/vol ratio of about 3.43 mm^−1^, whereas the conical leaves of *D. cooperi* have an sa/vol ratio of 1.65 mm^−1^ and the equally conical but shorter leaves of *D. harazianum* show an sa/vol ratio of about 1.78 mm^−1^ [47].

**Figure 10:**
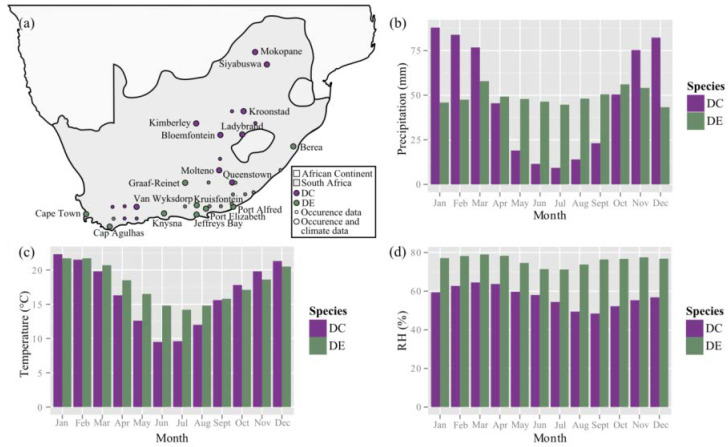
Occurrence and climate data of *D. cooperi* (DC) and *D. ecklonis* (DE). (**a**) Map of Southern Africa (light grey) and South Africa (dark grey) mapping the natural occurrences of *D. cooperi* and *D. ecklonis*. The larger dots mark locations from which climate data were acquired. (**b**) Monthly precipitation (mm), (**c**) monthly temperature (°C) and (**d**) monthly relative humidity (RH in %). The climate data for a given city originate from an average of 18–112 years on record for each city of occurrence of each species.

According to Griffith and Males [38], succulence generally tends to evolve in semi-arid environments, in which seasonal water deficits are common and rains “return after a relatively fixed period of time” rather than is the case in fully arid desert regions [38]. As mentioned above, a high periodicity of water availability characterizes regions of occurrence for *D. cooperi* (Figure 10) and seems to be the main reason for the observed different morphological-anatomical and biomechanical adaptations of *D. cooperi* and *D. ecklonis*. This raises the question as to whether the differences found within the framework of this study (Table 1 and Table 2) are also the basis for the differences in the self-sealing functions of the leaves of these two species.

Our results support this hypothesis insofar as the main difference between the species is the contraction of the incision region, which is more markedly pronounced in *D. cooperi* than in *D. ecklonis* (Figure 8). In *D. ecklonis*, the segment-specific contractions are, in general, less pronounced. In particular, a contraction of the apex, incision region and base of the damaged leaves can be detected for both the absolute and relative contraction of the leaves and are therefore decoupled from differences in the overall leaf length (Table 1), i.e., the size of individual leaf segments (Figure 7 and Figure 8, Figure A1 and Figure A2 in Appendix A). Both species show tendencies for higher contraction at lower RH levels (Figure 8). The latter is an indication that the injury-induced contraction is at least partly driven by a hydraulic mechanism (see [15] for details).

In principle, the movement of multicellular plants can be classified into movements caused by hydraulic mechanisms and movements caused by mechanical instabilities. The duration of movement can be plotted as a function of the tissue size, defined as the smallest macroscopic moving part. Generally, hydraulic movements are limited by the poroelastic timescale of water diffusion through a porous plant tissue (=fastest tissue swelling). Therefore, hydraulic movements including irreversible growth processes and reversible swelling and shrinking processes are typically slow. Movements caused by elastic instabilities are ultimately limited by the time scale for elastic wave propagation (=fastest elastic motion). Release of stored elastic energy or rapid geometric changes can speed up movements beyond limits imposed by simple hydraulic mechanisms [20,21]. Given a sealing time of 60 min and a leaf diameter of approximately 3 mm as a basis, the sealing movements of *Delosperma* leaves can be considered as being mainly driven by hydraulic processes. This is consistent with the conclusions of Klein et al. [15] who showed that hydraulic processes dominantly govern the self-sealing kinematics in *D. cooperi*. However, the dominant factor contributing to the observed differences in the self-sealing kinematics between the two studied species remains unclear, as does whether the pronounced contraction of the incision region in *D. cooperi* is linked to more efficient wound sealing.

In addition to movement attributable to hydraulic mechanisms, mechanical instabilities seem to be involved in movement in multicellular plants [20,21]. In this context Konrad et al. [4] have developed an analytical model based on elastic and visco-elastic deformations of intact and damaged leaves of *D. cooperi*. The centrosymmetric arrangement of five tissue layers has been abstracted as a shell structure of tissues alternatively under pre-tension (epidermis, net of vascular bundles, central vascular strand) and pre-compression (chlorenchyma, hydrenchyma). This embedded energy in terms of pre-stresses ensures an overall mechanical equilibrium in intact leaves, which is destroyed by damage, whereby the previously stored energy is released in the form of a movement until a new equilibrium is achieved and the wound edges are brought into contact (self-sealing) [4,16]. The extent of the relaxation movement of the circumferentially injured leaf depends markedly on the stiffness of the remaining intact tissue (central vascular strand and hydrenchyma) in the incision region at which epidermis, chlorenchyma and (pro parte) the net of vascular bundles are cut. Hence, we can assume that the central vascular strand is the mechanically dominant structure. With a median elastic modulus under tension of 32.80 MPa, the central vascular tissue of *D. cooperi* is approximately twice as flexible and therefore as extensible as that of *D. ecklonis*. Thus, the more pronounced contraction of the incision region of *D. cooperi* compared with *D. ecklonis* might mainly be linked to the significant mechanical differences in the elastic modulus of the central vascular strand; therefore, we can hypothesize that the central vascular strand in adult succulent leaves of *D. cooperi* is markedly more stretched and pre-tensioned than that in *D. ecklonis*.

Finally, some additional points need to be addressed and discussed that might be argued as having an additional effect on the efficiency of the observed self-sealing kinematics: (1) differences in the experimental setup, (2) effects of the different leaf geometries, (3) differences in the evaluation of the contraction, (4) differences in the size of the hydrenchyma cells and (5) differences in the number and shape of the epidermal bladder cells. We assume that, although these points are important, they do not have a dominant effect on the wound reaction. First, differences in the experimental setup of the leaf kinematic experiments might add to differences in the contractions measured for each species. Whereas *D. cooperi* leaves were positioned in an upright orientation by use of a sample holder, the leaves of *D. ecklonis* were positioned horizontally and thus retained their natural orientation. The resulting differences in the gravitational forces acting on the leaves might become visible in the contraction of the apex and base segments, which are always slightly more pronounced in *D. cooperi* (Figure 7 and Figure 8, Figure A1 and Figure A2 in Appendix A). However, gravitational forces cannot fully explain the strong contraction of the incision region of *D. cooperi* in comparison with that in *D. ecklonis* (Figure 7 and Figure 8, Figure A1 and Figure A2 in Appendix A). Second, differences in the geometry of the leaves can be neglected to a good approximation, as the leaves contract and do not bend. Third, the strong curvature of *D. ecklonis* leaves requires a polynomial fit in order for the observed leaf contraction to be calculated. This might lead to a small alteration from the natural contraction but still cannot explain the pronounced differences in the contraction of the incision region between the species. Fourth, differences exist in the hydrenchyma cells located in close proximity to the central vascular strand (Figure 2). In *D. ecklonis*, these hydrenchyma cells are much larger than those of *D. cooperi* and can expand into regions that might be included in the damage caused by the incisions applied during our experiments. Such an injury might result in the leakage of a notable amount of cell sap resulting in a relaxation of compressive stresses. Intraspecific, this could promote a more pronounced contraction in the incision region. Interspecific differences would, however, be ascribed to differences in the elastic modulus of the central vascular strand as described above. Fifth, the morphology and hydraulic function of epidermal bladder cells may lead to differences in the contraction of the leaves of the two species. In theory, a higher solute concentration in epidermal bladder cells of *D. cooperi* can result in a higher concentration of solutes in the periphery of the incision region as water evaporates [15]. This results in an osmotic gradient promoting a pronounced release of compressive stresses of parenchymatic cells in the incision region as water diffuses towards the incision surface and evaporates. In combination with higher flexibility of the central vascular strand, a more pronounced contraction results. Hence epidermal bladder cells could contribute to the more pronounced contraction of the incision region of *D. cooperi*. This would have to be tested by using other experimental approaches.

## 4. Materials and Methods

### 4.1. Plant Material

Plants of *D. cooperi* and *D. ecklonis* from old stocks of the Botanic Garden Freiburg (University of Freiburg, Freiburg, Germany) were kept in the greenhouses of the Botanic Garden under standard conditions. Individual plants were selected according to the following criteria: (1) comparable ontogenetic stages of the leaves, (2) identical size of flowering pot, (3) comparable age of the plants and (4) absence of injuries from the respective leaves. For each experiment, ten leaves of ten plants were selected per species (*n* = 10).

### 4.2. Occurrence and Climate Data

The occurrence data of the natural habitats of *D. cooperi* and *D. ecklonis* were acquired from the Bolus Herbarium Specimens Collection found on the website of the Global Biodiversity Information Facility [48]. Only complete records of occurrences in South Africa were considered, as *Delosperma* species are native to this region [35,49]. The geographic coordinates of each dataset were used for determining the location of occurrence and its altitude and subsequently for identifying a city in close proximity to the location of occurrence (max. distance: 50 km). Climate data for the natural habitats of each species were acquired from climate-data websites (en.climate-data.org and weatherbase.com) by using ten cities near the natural occurrences of each species. The climate data for a particular city of occurrence for each species was hereby given as an average of 18–112 years on record. Data concerning the monthly temperature (°C), precipitation (mm) and relative humidity (RH in %) were visualized in grouped bar plots by using the *ggplot2* package of the open source software *R* [50,51].

*D. cooperi* and *D. ecklonis* are both native to South Africa (Figure 10a). However, *D. ecklonis* dominantly occurs in coastal regions with mean altitudes of 283.1 ± 274.4 m.a.s.l. (Table A1 in Appendix B). Growing areas of *D. cooperi*, on the other hand, are located at or near the Drakensberg Mountains with a mean altitude of 1419.2 ± 292.6 m.a.s.l. (Table A1 in Appendix B). Although the annual mean precipitation (in mm) does not differ greatly between the natural occurrences of both species (Table A1 in Appendix B), drastic seasonal differences can be detected: The precipitation is more evenly distributed throughout the year for the natural habitat of *D. ecklonis*, whereas *D. cooperi* habitats are characterized by seasonal fluctuation of alternating wet summer (Nov.–Mar.) and dry winter (Jun.–Aug.; Figure 10b) periods. The annual mean temperature only differs by approximately 1 °C between both species (Table A1 in Appendix B), with habitats of *D. ecklonis* being warmer. However, the temperature is more evenly distributed throughout the year for *D. ecklonis*, as winter months (Jun.–Aug.) are warmer and summer months (Nov.–Mar.) are colder when compared with those for *D. cooperi* (Table A1 in Appendix B and Figure 10c). In addition, the habitats of *D. ecklonis* are more humid and the daily fluctuations of the relative humidity (RH) are only in the range of approximately 20% (Figure 10d, Table A1 in Appendix B). This differs from *D. cooperi* habitats, which show daily fluctuations of RH in the range of 40% (Table A1 in Appendix B). Thus, *D. ecklonis* is native to oceanic climates with a Köppen-Geiger climate classification of Cfb (“warm temperate climate, fully humid”; [45,46]), whereas *D. cooperi* commonly occurs in regions with a climate classification of Cwb (“warm temperate climate with dry winter”; [45,46]).

### 4.3. Leaf Morphometry

**Figure 11:**
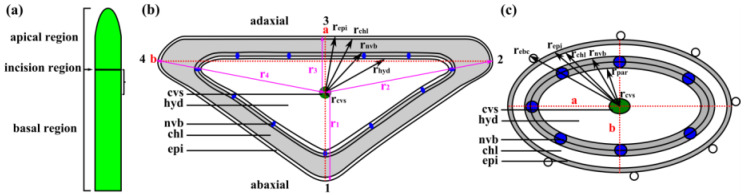
Schematic representation of a leaf (**a**), which was divided by the incision region into the apical region equaling one-third and the basal region equaling two-thirds of the total leaf length. Schematic drawings of transverse sections of leaves of (**b**) *D. ecklonis* and (**c**) *D. cooperi* (based on investigations in [15]). The epidermis (epi), chlorenchyma (chl, grey), net of peripheral vascular bundles (nvb, blue), hydrenchyma (hyd) and central vascular strand (cvs, green) have been simplified to five consecutive tissue layers. The thickness of each tissue layer was calculated for *D. cooperi* by measurements along the diameters a and b (red dotted lines) and for *D. ecklonis* by measurements along the pink lines r_1_–r_4_ (see Appendix C).The tissue arrangement within a transverse section of a leaf of *D. ecklonis* or *D. cooperi* can be simplified to five consecutive layers with the central vascular strand (cvs) being the inner most layer followed by the hydrenchyma (hyd), the peripheral net of vascular bundles (nvb), the chlorenchyma and then the epidermis (epi) as the outermost layer (Figure 11b,c).

Ten fresh leaves taken from ten plants, respectively, were first measured for total length and diameter by using a digital caliper (Absolute Digimatic No. CD-15CPX, Mitutoyo Corporation, Kawasaki, Japan). Total length was measured in order to determine the position in which the leaf was to be divided into an apical region equaling one-third of total leaf length and a basal region equaling two-thirds of total leaf length (Figure 11a). At the boundary between the apical and basal regions, artificial wounds were applied in leaf kinematics experiments. The leaf diameters were measured along the red dotted lines a and b in Figure 11b.

For *D*. *cooperi,* the radius of each tissue layer was calculated based on the measurements along the diameters a and b (red dotted lines in Figure 11c). The dimension of each tissue layer of the triangular leaf geometry in *D. ecklonis*, however, was measured along r_1_–r_4_ (pink lines in Figure 11b), whereby r_1_, r_2_ and r_3_ were the interior angle bisectors and r_4_ was the side bisector. The thickness of each tissue layer was calculated from a given tissue radius that was subtracted by the radius of the adjacent inner tissue. Further details for the calculation of the thickness of tissue layers for *D. ecklonis* are given in Appendix A and for *D. cooperi* in [15].

The geometrical dimensions of *D. ecklonis* and *D. cooperi* were measured on embedded cross-sections of the leaves. However, the embedding procedure results in shrinkage of tissues. This shrinkage mainly affects hydrenchyma and chlorenchyma tissues and can be calculated from the embedded cross-sections as the percentage shrinkage (γ in %) relative to the fresh condition of a leaf (see Appendix D). For the sake of completeness, the percentage shrinkage of *D. ecklonis* was calculated and documented for a construction of a relatively accurate numerical model of a “fresh” leaf according to procedures applied in [15].

### 4.4. Leaf Anatomy

#### 4.4.1. Light Microscopy

Subsequent to the leaf kinematics experiment, a leaf fragment with an axial length of 5 mm was cut from the basal leaf region located directly underneath the incision region (Figure 11a). Each of the ten *D. ecklonis* leaf fragments were then fixed, dehydrated and embedded according to the same protocol as described for *D. cooperi* in [15]. Contrast staining of the thin sections was performed with toluidine blue (0.05% toluidine blue in distilled water), which stains unlignified primary cell walls in dark blue and lignified secondary cell walls in light blue. The examination and image acquisition of thin sections was performed with an Olympus BX61 microscope (Olympus Corporation, Tokyo, Japan) equipped with a DP71 camera module. Morphometric parameters were determined from each thin section according to [15,16] by using the image analysis software ImageJ 1.440 h [52].

#### 4.4.2. Scanning Electron Microscopy

Leaf tips were cut using a razor blade and immediately placed in a dehydration series of methanol solutions from 30% to 100% methyl alcohol [53]. The samples were then critical-point dried (CPD 030, Bal-Tec AG, Balzers, Lichtenstein) with acetone as the exchange medium and by the use of CO_2_ before being mounted on aluminum stubs with conductive double-sided adhesive tabs (Plano GmbH, Wetzlar, Germany) and aqueous conductive silver (Mikrotechnik Dr. Hert GmbH, Munich, Germany). After being sputter-coated for 120 s with gold (Cressington Sputter Coater, 108auto, EO Elektronen-Optik-Service GmbH, Dortmund, Germany), the samples were examined in a scanning electron microscope (Leo 435 vp, Leica, Leo Electron Microscopy Ltd. Cambridge, England).

#### 4.4.3. Magnetic Resonance Imaging

Leaves were carefully detached from the plant by using a razor blade. The cut-surface was wrapped with paper towels infiltrated with a 10 mM solution of MnCl, which was used as a contrast agent to highlight the vascular system. The leaf tip was then imaged using a 7 T Bruker Biospec 70/20 small animal scanner and a two cm surface cryo-coil. Imaging was performed with a 3D FLASH sequence with the following parameters: *D. cooperi*: TR = 8 ms, TE = 2.3 ms, flip angle of 30°, 32 Averages, voxel size of 106 × 113 × 117 μm^3^, total acquisition time of 20 min; *D. ecklonis*: TR = 10 ms, TE = 3 ms, flip angle of 60°, 16 Averages, voxel size of 78 × 78 × 78 μm^3^, total acquisition time of 35 min. The parameters differed between the species to compensate for the low tissue contrast between the hydrenchyma tissue and vascular system in *D. ecklonis*. In *D. ecklonis*, hydrenchyma cells, particularly those located close to the central vascular strand, are extremely large, which leads to the bright appearance of this tissue in comparison with the chlorenchyma tissue, which has darker pixel intensity values.

The acquired image raw data was post-processed using 3D Slicer, an open source software (v. 4.3.1; [54]), for developing (quasi) 3D data representations of the leaf tips of *D. cooperi* and *D. ecklonis*. The outer geometry of the leaves was first isolated from the image raw data (nii. files) by using a threshold segmentation tool and the resulting segmentation image was then manually corrected using the paintbrush segmentation tool. Subsequently, the vascular system of the leaves was isolated manually using the paintbrush tool. The image segmentation was relatively difficult for *D. ecklonis* because of the low tissue contrast between the hydrenchyma and vascular tissues, as reflected in the results.

### 4.5. Leaf Biomechanics

Tensile tests of entire leaves and individual tissues (epidermis, central strand of vascular bundles) were performed on a modified custom-made micro-tensile-testing device (Technical Workshop, Institute of Biology II/III, University of Freiburg, Freiburg, Germany). Elastic moduli and tensile strength were calculated as described in [16]. The elastic modulus of the hydrenchyma and chlorenchyma was calculated by using an equation that was suggested by Nilsson et al. [31] and that describes the linear relationship between elastic modulus and turgor (for details, see [16]). As in Speck et al. [16], the turgor of individual parenchymatous cells was measured at various measuring depth in the leaf with a cell pressure probe (Lehner GmbH Sensor Systeme, Kirchheim/Teck, Germany).

### 4.6. Leaf Kinematics

For reasons of comparability, an experimental setup and approach similar to the work of Klein et al. [15] was chosen to analyze the self-sealing kinematics of twenty *D. ecklonis* leaves in response to a ring incision (Figure 11a). However, leaves of *D. cooperi* are glabrous and straight and do not show a curvature, whereas *D. ecklonis* leaves are pubescent and recurved. Both the hairs and the curvature of *D. ecklonis* leaves prohibit the use of a specimen holder, such as was used for the kinematic experiments of the leaves of *D. cooperi* [15]. Hence, *D. ecklonis* leaves were analyzed in a horizontal orientation without a specimen holder and not in an upright posture with specimen holder, as was the case for *D. cooperi*. The branches bearing the leaves under analysis were fixed in position by using bamboo (kebab) sticks and tape in order to avoid biasing the leaf movements with movements of the branch.

A custom-built laboratory bench phytochamber (Technical Workshop, Institute of Biology II/III, University of Freiburg, Freiburg, Germany) was used to set the average chamber temperature to 28 °C in all experiments (Figure 6a). A built-in ultrasonic fogger (Fogger XL, Trixie GmbH & Co. KG, Tarp, Germany) regulated the relative humidity (RH) within the phytochamber to approx. 44% RH or 60% RH in order to perform ten leaf kinematic experiments for each RH. A data logger (MSR145b, MSR Electronics GmbH, Seuzach, Switzerland) live-recorded the chamber temperature and RH values throughout all experiments. In addition, a custom-built mounting device (Technical Workshop, Institute of Biology II/III, University of Freiburg, Germany) was developed for positioning two identical Basler cameras (acA2040–90um, Basler AG, Ahrensburg, Germany) with CCTV lenses (LM35HC, Kowa Optical Products Co. Ltd., Nagoya, Japan) at a right angle equidistant from the center of the phytochamber. The cameras were used to live-image the self-sealing kinematics of each leaf from the front and the side simultaneously. Images acquired by the frontal camera were examined to ensure a maximum error of 5% attributable to perspective imperfections (see [15] for further details).

The leaves were preselected for similar ontogenetic stage (approx. third leaf below the apical meristem), not having a pronounced curvature and with an approximate length of 27 mm (see Table 1) to minimize the effect of phenotypic plasticity of the leaves [35,43] on the motion analyses. The length of a leaf and the minor and major leaf diameters at the apex, center and base were measured in the X and Z directions (Figure 6c) by using a digital calliper. Subsequently, a white gloss paint marker (edding 780 creative, edding International GmbH, Ahrensburg, Germany) was used to apply five points along the longitudinal axis and the upper edge of a selected leaf as the main markers for motion analysis; these points highlighted the apex (p1), the regions two millimeter above (p5) and below (p9) the incision, the base (p17) and half the distance between points p9 and p17 (p13) of the leaf (Figure 6c). Ring incisions with a standardized depth of 0.8 mm were applied at the chosen incision region (Figure 11a) by using a custom-built cutting device (Technical Workshop, Institute of Biology II/III, University of Freiburg, Freiburg, Germany). Directly after the incision, plants were placed into the phytochamber with the injured leaf in alignment to a lateral and frontal camera (Figure 6a). The pre-imaging preparation, from the application of the ring incision to the beginning of image acquisition, took on average less than three minutes.

The acquisition software *pylon Viewer* (version 5.0.5.8999, Basler AG, Ahrensburg, Germany) was used to record the leaf kinematics of *D. ecklonis* for one hour at a capturing rate of 1/6 Hz. The recording images were then transferred into the software *Photron FASTCAM Viewer* and *Photron FASTCAM Analysis* (PFV version 3.6.5.0, PFA version 1.3.2.0, Photron Europe, West Wycombe, Buck, UK) to track the XY positions of marker points (p1 to p17) on a frame-by-frame basis (Figure 6b) in order to determine a segment-specific (“apex”, “incision” and “base” in Figure 6c) absolute contraction. The determination of the segment-specific absolute contractions differed slightly between *D. ecklonis* and *D. cooperi* [15] because of the different leaf curvatures of the species. For *D. cooperi* (straight leaves), the tracking of four points per leaf was sufficient. Here, the calculation of the absolute contractions originated directly from the changes in tracking point spacing (Y-axis). For *D. ecklonis* (recurved leaves), the tracking of seventeen points (five points were edding markers and the rest were virtually determined markers) per leaf was necessary. Afterwards, a polynomial fit was applied to the seventeen tracking points allowing the segment-specific determination of the absolute contractions in *D. ecklonis* by arc length analysis.

Absolute contraction (ε_i_(t)):

ε_i_(t) = (L_i_(t) − L_i_(t_0_))/L_i_(t_0_) (1)

i = segment identifier; L = segment length, Y-axis spacing for *D. cooperi* [15]; arc length spacing for *D. ecklonis*; t_0_ = beginning of measurement; t = time point during experiment;

Relative contraction (ε_i_^*^(t)):

ε_i_^*^(t) = ε_i_(t)∙(L_i_(t)/L_total leaf_(t)) (2)

i = segment identifier; L = segment length, Y-axis spacing for *D. cooperi* [15]; arc length spacing for *D. ecklonis*; t = time point during experiment;

The relative contraction considers the differences of the mean leaf length of both species. In general, a total of twenty leaves per species was analyzed according to their self-sealing kinematics (see [15] for *D. cooperi*). Ten of these twenty leaves per species were tested at low RH levels (*D. cooperi*: 44.0%; *D. ecklonis*: 43.6%). The remaining ten leaves per species were tested at high RH levels (*D. cooperi*: 59.6%; *D. ecklonis*: 57.3%).

### 4.7. Statistics

Raw data are provided in supplements. The software *GNU R* 3.5.2 including the additional packages *ggplot2* and *psych* was used for statistical analyses [50,51,55]. Descriptive statistical analyses were carried out to compare the habitat-specific climate data and the morphometrical and biomechanical parameters of the succulent leaves of *D. cooperi* and *D. ecklonis*. If not stated otherwise, parametric data were represented by mean values ± standard error, whereas non-parametric data were shown as median values with respective standard error. Shapiro’s and Levene’s tests were applied to check for normal distribution and homoscedasticity of variances, respectively. Furthermore, the statistical analyses of the non-parametric leaf kinematics data were performed by using Scheirer-Ray-Hare tests, i.e., two-way ANOVA’s on rank-transformed data, in combination with pairwise Wilcoxon rank sum post-hoc tests with adjusted *p*-values (using Bonferroni’s method). For additional statistical comparisons of the biomechanical properties between *D. cooperi* and *D. ecklonis* leaves, we conducted Wilcoxon Mann–Whitney signed rank tests. Leaf morphometric parameters were statistically analyzed using unpaired and paired two-sample t-tests in cases of inter- and intraspecific comparisons, respectively. Finally, the relationship between the turgor pressure and the respective cell position within a given leaf was evaluated using Spearman’s rank-order correlation analysis. Levels of significance were: *p* > 0.05: not significant (n.s.); *p* ≤ 0.05: significant (*); *p* ≤ 0.01: very significant (**); *p* ≤ 0.001: highly significant (***).

## 5. Conclusions

The genus *Delosperma* is a highly suitable group of biological archetypes that might help to understand the underlying functional principles behind self-sealing kinematics in succulent leaves. The genus includes a group of species that reflect transitions between differing ecological niches by recent diversification [9,10]. This is manifested in the pronounced phenotypic plasticity of *Delosperma* leaves, as mentioned by Hartmann [35,43], and has allowed a relatively fast adaptation to changing climatic conditions. In this context the key innovation of an efficient and rapid self-sealing of external injuries may have played a crucial role during biological evolution. The presented morphological-anatomical and biomechanical study comparing the self-sealing kinematics of two *Delosperma* species native to different ecological niches supports our hypothesis of a self-sealing mechanism dependent on water availability. Although such an investigation can be classified as basic research in the field of botany, scientists from other scientific disciplines may be inspired for a technical application with self-sealing function. Within the framework of the biomimetic bottom-up process [56], Yang et al. [28] developed a self-repairing material with shape memory effect inspired by the functional principles found in *Delosperma* leaves. In recent years, more and more bioinspired and biomimetic self-repairing material systems have been developed for the technical field [8]. Ultimately, this is tantamount to a paradigm shift in damage control, namely from damage prevention to damage management [8].

## Figures and Tables

**Table 1:** Morphometric data of the leaves of *D. cooperi* and *D. ecklonis*, showing the mean and the standard error (s.e.) of the total leaf length (L), the leaf diameters (d) a and b for fresh leaves (f) and the tissue thicknesses (t) of the epidermal bladder cells (ebc), the epidermis (epi), the chlorenchyma (chl), the net of vascular bundles (nvb), the hydrenchyma (hyd) and the central vascular strand along the minor (a) and major (b) diameter of a leaf. The respective sample size (*n*) is also given. The results of the interspecific statistical analyses are based on two-sample *t*-tests and are shown as test statistic (*t*), degree of freedom (d.f.) and statistical significance. The statistical significance of intraspecific comparisons is given as super-scripts behind the respective sample means (without test statistic and degree of freedom) based on paired two-sample *t*-tests. The data for *D. cooperi* were acquired during experiments conducted by Klein et al. [15].

	*D. ecklonis*		*D. cooperi*		Interspecific Statistics		
	mean ± s.e.	*n*	mean ± s.e.	*n*	*t*	d.f.	Significance
L_f_ (mm)	27.4 ± 1.3	20	45.5 ± 0.6	10	−12.7	26.1	***
d_a, f_ (mm)	2.5 ± 0.1 ^***^	20	3.4 ± 0.1 ^***^	10	−7.2	27.0	***
d_b, f_ (mm)	5.4 ± 0.2 ^***^	20	3.0 ± 0.03 ^***^	10	13.1	20.3	***
t_ebc, a_ (µm)	82.6 ± 5.5 ^ns^	20	249.5 ± 11.1 ^ns^	20	−14.1	27.9	***
t_ebc, b_ (µm)	84.3 ± 3.7 ^ns^	20	258.4 ± 9.5 ^ns^	20	−16.3	24.5	***
t_epi, a_ (µm)	24.4 ± 1.9 ^ns^	20	40.1 ± 2.4 ^ns^	20	−5.1	36.0	***
t_epi, b_ (µm)	26.7 ± 1.9 ^ns^	20	40.2 ± 3.2 ^ns^	20	−3.6	31.1	***
t_chl, a_ (µm)	364.3 ± 19.6 ^***^	20	524.9 ± 22.1 ^ns^	20	−5.4	37.5	***
t_chl, b_ (µm)	775.3 ± 25.0 ^***^	20	566.6 ± 16.7 ^ns^	20	7.0	33.1	***
t_nvb, a_ (µm)	55.4 ± 2.3 ^*^	20	64.4 ± 1.8 ^ns^	20	−3.0	36.2	**
t_nvb, b_ (µm)	63.2 ± 3.0 ^*^	20	65.6 ± 1.8 ^ns^	20	−0.7	31.5	ns
t_hyd, a_ (µm)	443.7 ± 30.8 ^***^	20	586.8 ± 28.8 ^ns^	20	−3.4	37.8	**
t_hyd, b_ (µm)	1450.6 ± 32.8 ^***^	20	671.2 ± 25.0 ^ns^	20	18.9	35.5	***
t_cvs, a_ (µm)	50.9 ± 2.9 ^***^	20	54.9 ± 1.1 ^*^	20	1.6	37.3	ns
t_cvs, b_ (µm)	66.9 ± 2.2 ^***^	20	60.6 ± 2.6 ^*^	20	−1.7	27.9	ns

* significant at *p* ≤ 0.05; **, significant at *p* ≤ 0.01; ***, significant at *p* ≤ 0.001; ns, not significant at *p* > 0.05.

**Table 2:** Mechanical properties and additional mechanically important characterization of entire leaves and single tissue layers of *D. ecklonis* and *D. cooperi*. The median value (med), the standard error (s.e.) and the sample size (*n*) for each parameter are given. The results of the inter- and intraspecific statistical analyses are both based on Wilcoxon-Mann-Whitney signed rank tests. The statistical significance of intraspecific comparisons is given as super-scripts behind the respective sample medians. Values for *D. ecklonis* are taken from [33,34]. Values for *D. cooperi* are taken from [16].

	*D. ecklonis*			*D. cooperi*			Ratio		Significance
	med ± s.e.	*n*		med ± s.e.	*n*		med_ecklonis_/med_cooperi_		
Elastic Modulus in Tension (MPa)									
Leaf	1.21 ± 0.11	14		0.72 ± 0.14	18		1.68		***
Central Vascular Strand	63.68 ± 7.52	9		32.80 ± 5.96	8		1.94		**
Epidermis (trans.)	2.88 ± 0.40 ^ns^	6		3.62 ± 0.27 ^ns^	10		0.80		*
Epidermis (long.)	3.51 ± 1.21 ^ns^	8		5.27 ± 0.85 ^ns^	10		0.67		ns
Hydrenchyma ^†^	0.26	‒		0.23	‒		1.13		‒
Chlorenchyma ^†^	0.26	‒		0.27	‒		0.96		‒
Tensile Strength (MPa)									
Leaf	0.16 ± 0.01	14		0.09 ± 0.01	13		1.78		***
Central Vascular Strand	10.99 ± 0.70	9		8.80 ± 0.76	8		1.25		**
Epidermis (trans.)	0.58 ± 0.10 ^ns^	6		1.25 ± 0.07 ^*^	10		0.46		**
Epidermis (long.)	0.70 ± 0.26 ^ns^	8		1.54 ± 0.11 ^*^	10		0.45		**
Poisson’s Ratio									
Leaf	0.35 ± 0.03	14		0.29 ± 0.03	18		1.21		*
Turgor (MPa)									
Hydrenchyma and Chlorenchyma	0.05 ± 0.002	36		0.04 ± 0.003	44		1.25		ns

^†^ Calculated by using the equation postulated by Nilsson et al. [33] by using median values (for detailed descriptions see [16]); *, significant at *p* ≤ 0.05; **, significant at *p* ≤ 0.01; ***, significant at *p* ≤ 0.001; ns, not significant at *p* > 0.05, ‒ no values applicable.

**Table 3:** Summary of species specific characteristics of *D. ecklonis* and *D. cooperi*.

Feature	*D. ecklonis*	*D. cooperi*
**Habitat and Climate**		
Habitat	coastal regions in South Africa	highland regions in South Africa
Köppen-Geiger Climate Classification	Cfb = oceanic climate (warm temperate climate, fully humid)	Cwb = dry-winter subtropical highland climate (warm temperate climate with dry winter)
**Morphology and Anatomy**		
Plant Habitus	branched shrub, erect branches, height: 129.6 ± 27.2 mm, diameter: 149.5 ± 57.0 mm	branched shrub, decumbent, height: 68.0 ± 13.7 mm, diameter: 150.0 ± 44.0 mm
Leaf Shape	triquetrous	semi-terete to terete
Leaf Morphology	pubescent, recurved	straight
Leaf Length	27.4 ± 26.5 mm	45.5 ± 1.9 mm
Epidermal Bladder Cells	thickened basally but grow out to slim trichomes distally	rounded and papillose, large and densely packed
Hydrenchyma	mean values of cell diameter and mean aspect ratio of cells considerably higher than in *D. cooperi*	
**Biomechanics (Elastic Modulus/Tensile Strength)**		
Entire leaf, central vascular strand	considerably stiffer and more rigid than in *D. cooperi*	
Epidermis		considerably stiffer and more rigid than in *D. ecklonis*
**Further Mechanically Important Parameters**		
Poisson’s Ratio	significantly higher than in *D. cooperi*	
Turgor	slightly higher than in *D. cooperi*	
**Leaf Kinematics**		
Absolute and relative contractions (esp. incision region) independent of the given relative humidity (RH) level		more pronounced than in *D. ecklonis*

## Supplementary Materials

Supplementary materials can be found at https://www.mdpi.com/1422-0067/21/16/5768/s1 or at Zenodo: Supplementary Video 1: 10.5281/zenodo.3885166; Supplementary Video 2: 10.5281/zenodo.3885174; Data availability file: 10.5281/zenodo.3885176.

## Appendix B. Climate-Specific Data

**Table A1:** Climate data acquired on the basis of occurrence data of the natural habitats of *D. cooperi* and *D. ecklonis*. The mean value, standard deviation (sd), minimal (min) and maximal (max) values and number of acquired climate data (*n*) are given for the altitude of occurrence (m), the annual mean precipitation (mm), the annual mean temperature (°C), the annual minimal and maximal temperature (°C), the annual relative humidity (RH in %) and daily fluctuations of the RH (morning and evening RH) of each species. The climate data for a given city originates from an average of 18–112 years on record for each city of occurrence of each species.

	*D. ecklonis*					*D. cooperi*				
	Mean	sd	Min	Max	*n*	Mean	sd	Min	Max	*n*
Altitude (m)	283.1	274.4	10.0	838.2	10	1419.2	292.6	953.0	1920.2	10
Annual mean prec. (mm)	601.7	176.1	332.0	1009.4	10	585.5	77.0	420.0	713.4	10
Annual mean temp. (°C)	17.9	1.1	16.0	20.4	10	16.5	1.9	12.9	20.0	10
Max. temp. (°C)	21.6	1.1	20.0	23.2	6	24.0	1.9	21.0	28.0	10
Min. temp. (°C)	13.0	1.0	11.5	14.5	6	8.9	2.3	5.2	12.3	10
Annual RH (%)	75.9	3.3	69.0	80.0	8	57.0	2.5	52.1	59.7	8
Morning RH (%)	86.5	0.5	86.0	87.0	2	73.5	6.5	67.0	80.0	2
Evening RH (%)	68.0	4.0	72.0	64.0	2	32.5	1.5	31.0	34.0	2

## Appendix C. Calculation of Radii of Individual Tissue Layers

Leaves of *D. ecklonis* have a triangular cross-sectional area in which the respective tissue layers are calculated on the basis of measurements along the pink lines r_1_–r_4_ (Figure 11b), where r_1_, r_2_ and r_3_ are the interior angle bisectors and r_4_ is the side bisector. The distance of the outermost cell of a given tissue from the center of the central vascular strand (cvs) is defined as the “equivalent tissue radius”, which is subsequently used to calculate the tissue thickness (t) of a given tissue by subtracting it from the “equivalent radius” of the adjacent inner tissue. The “equivalent tissue radii” r_1_ and r_3_ are averaged to “radius” r_a_ and the “equivalent tissue radii” r_4_ and r_2_ are averaged to “radius” r_b_ for each given tissue. The “radii” and tissue thicknesses are determined in this manner to allow comparisons with the major (a) and the minor (b) radii of *D. cooperi* measured according to Klein et al. [15] as indicated in Figure 11c (see results in Table 1).

The tissue thicknesses of the peripheral net of vascular bundles and epidermal bladder cells are determined differently. The peripheral net of vascular bundles is visible as isolated vascular bundles within the periphery of a transverse section of a leaf. The tissue thicknesses of three individual peripheral bundles were measured per “radial” lines r_1_–r_4_ and averaged. The parenchyma tissue located between the peripheral vascular bundles is split in half (as indicated in Figure 11b) and the outer portion is assigned to the chlorenchyma, while the inner half is assigned to the hydrenchyma. The basally thickened region of the epidermal bladder cells was measured for two bladder cells located in close proximity to the lines r_1_–r_4_ and averaged.

## Appendix D. Percentage Shrinkage

The dimensions of leaves and tissues of *D. ecklonis* and *D. cooperi* were measured on embedded cross-sections of the leaves. However, the embedding procedure results in shrinkage of parenchymatic tissues relative to the fresh condition of a leaf. Thus, the ten leaf samples for the preparation of thin sections were additionally used in order to determine the percentage shrinkage (γ) of a leaf sample during the embedding process. The percentage shrinkage (γ_a_ and γ_b_ in %) was calculated for the leaf diameters a and b (Figure 11b,c). The percentage shrinkage was calculated and documented for a future construction of a relatively accurate numerical model of a “fresh” leaf according to procedures applied in Klein et al. [15].

The embedding of leaf samples of *D. ecklonis* leads to a reduction of the transverse diameter a (see Figure 11) at the incision region of 11.6 ± 6.1%. The diameter b, however, increases to about 7.4 ± 6.5% in relation to the fresh condition of the leaf (from 4.3 ± 0.3 mm to 4.6 ± 0.2 mm; see Table A2).

**Table A2:** Percentage shrinkage (γa and γb in %) along major and the minor radial diameters a and b of the cross-section area of an embedded transverse section relative to the fresh condition of a leaf. The mean values with standard deviations (sd), the minimum (min) and maximum (max) values of the major and the minor radial diameters at the incision region (a_1/3_ and b_1/3_) and the percentage shrinkage (γ_a_ and γ_b_) for ten samples (*n* = 10) are given. Values for the percentage shrinkage of *D. cooperi* leaves are taken from Klein et al. [15] and given for comparison.

*D. ecklonis*							*D. cooperi*					
	Fresh		Embedded		Shrinkage		Fresh		Embedded		Shrinkage	
	a_1/3_	b_1/3_	a_1/3_	b_1/3_	γ_a_ (%)	γ_b_ (%)	a_1/3_	b_1/3_	a_1/3_	b_1/3_	γ_a_ (%)	γ_b_ (%)
mean	2.2	4.3	2.0	4.6	11.6	−7.4	3.4	3.0	2.9	2.6	14.8	13.9
sd	0.2	0.3	0.2	0.2	6.1	6.5	0.2	0.1	0.3	0.2	4.0	5.0
min	1.9	3.8	1.6	4.3	−0.8	−18.5	3.1	2.8	2.5	2.2	8.8	4.0
max	2.7	4.7	2.3	5.0	23.7	3.4	4.0	3.1	3.6	2.9	22.1	19.6
